# The Association of Type 2 Diabetes and Hypothyroidism with the Composition and Antioxidant Properties of Human Milk: A Cross-Sectional Study

**DOI:** 10.3390/nu18213713

**Published:** 2026-11-09

**Authors:** Agnieszka Dombrowska-Pali, Agnieszka Chrustek, Dorota Olszewska-Słonina, Maciej W. Socha

**Affiliations:** 1 Department of Perinatology, Gynecology and Gynecologic Oncology, Faculty of Health Sciences, Collegium Medicum in Bydgoszcz, Nicolaus Copernicus University, Łukasiewicza 1, 85-821 Bydgoszcz, Poland; 2 Department of Pathobiochemistry and Clinical Chemistry, Faculty of Pharmacy, Collegium Medicum in Bydgoszcz, Nicolaus Copernicus University, M. Curie-Skłodowskiej 9, 85-094 Bydgoszcz, Poland; 3 2nd Department of Gynecology and Obstetrics, Medical University of Gdańsk, 80-210 Gdansk, Poland

**Keywords:** human milk, nutrition, antioxidants, diabetes, thyroid diseases

## Abstract

Background: Thyroid disorders and diabetes are among the most common endocrine and metabolic conditions affecting pregnant women. It is important to note that adequate serum thyroid hormone levels are essential for proper lactation. Diabetes, in turn, is associated with delayed lactogenesis, especially in cases of poor glycemic control. It should be emphasized that breastfeeding is globally recognized as the safest and most effective method of feeding infants. Human milk has a variable nutritional composition in distinct phases: colostrum, transitional milk, and mature milk. Methods: The present observational cross-sectional study included 208 breastfeeding women between 3 and 12 months postpartum: 79 women with hypothyroidism, 53 women with type 2 diabetes, and 76 healthy controls. An analysis was conducted on a sample of human milk to determine its basic nutritional composition, cortisol concentration, and antioxidant status. This analysis utilized DPPH and FRAP assays, which are methods employed to assess the antioxidant capacity of a sample. The basic composition of human milk was analyzed using the MIRIS HMATM analyzer (Uppsala, Sweden). Cortisol levels were determined using an immunoenzymatic method. Results: In unadjusted analyses, milk fat content was higher in women with hypothyroidism and lower in women with type 2 diabetes compared with the control group. However, these differences were no longer statistically significant after adjustment for maternal and clinical covariates in multiple regression analyses. A lower reduction in the DPPH radical was shown in the milk of women with type 2 diabetes (13.8%) and in the milk of women with hypothyroidism (29%) compared to the control group. In the group of women with type 2 diabetes, cortisol levels positively correlated with the protein content and caloric content of milk. It was also noted that BMI in this group was significantly associated with the caloric content of human milk. Conclusions: These findings demonstrate differences in selected characteristics of human milk among the studied groups; however, their clinical relevance for infant health cannot be determined from the present study, as infant growth, body composition, and developmental outcomes were not assessed. Further prospective studies incorporating infant outcomes are needed to determine the clinical significance of these observations.

## 1. Introduction

Human milk (HM) provides infants with essential nutrients and contains numerous immunological and bioactive components that contribute to their growth, maturation, and development [1]. Moreover, human milk is considered a “dynamic biological secretion,” the composition of which depends on many factors, such as the gestational age at delivery, maternal diet, and pathologies related to the mother’s health [2]. In particular, maternal metabolic health, including diabetes, may alter the macronutrient profile of human milk [3]. A 2025 systematic review and meta-analysis demonstrated differences in macronutrient content and highlighted emerging evidence of altered milk lipid profiles in mothers with diabetes. Furthermore, differences in human milk composition between diabetic women were not associated with factors such as age and BMI [4]. Thyroid disorders are another factor that may influence the composition of human milk. Previous studies indicate that altered thyroid function can influence milk production as well as its biochemical composition. Hormonal disturbances observed in the course of these diseases may lead to changes in the content of certain nutrients and biologically active factors, but available research results are not always clear. Evidence for the effect of maternal hypothyroidism on breast milk composition remains limited. Experimental studies conducted in animal models indicate that untreated hypothyroidism during lactation can lead to delayed growth of offspring, reduced oxytocin secretion, weakened prolactin signaling, accelerated mammary gland involution, and changes in milk composition, including reduced triglyceride (TG) and carbohydrate concentrations [5,6]. Clinical studies have shown that hypothyroidism during pregnancy is associated with changes in the composition of both colostrum and mature milk. Among other things, reduced concentrations of proteins involved in macronutrient metabolism, increased immunoglobulin content in colostrum, and reduced fat and protein content in mature milk have been observed [7,8]. Importantly, the described differences also persisted in women receiving thyroid hormone replacement therapy. Despite the fact that prior studies have assessed the macronutrient composition of human milk in women with metabolic disorders, relatively less attention has been paid to its endocrine and antioxidant properties. Milk cortisol is an important bioactive hormone that may participate in the regulation of infant metabolism, stress regulation, immune function, and neurodevelopment. Likewise, oxidative balance in human milk is increasingly recognized as an important determinant of milk quality, as excessive oxidative stress may affect the stability and biological activity of nutrients and other bioactive compounds. DPPH radical scavenging activity and ferric reducing antioxidant power (FRAP) are complementary methods widely used to assess antioxidant capacity by evaluating different mechanisms of antioxidant action. Despite growing interest in the effects of maternal metabolic diseases on human milk, data on cortisol concentration and antioxidant status in the milk of women with hypothyroidism and type 2 diabetes remain limited. This lack of evidence represents a significant gap in the existing literature and is the rationale for the present study.

Recent advances in metabolomics and other omics technologies have substantially improved our understanding of the biological complexity of human milk. Recent studies have demonstrated that maternal conditions such as obesity, diabetes, hypothyroidism, and other components of metabolic syndrome are associated with alterations in the metabolomic and bioactive profiles of human milk. Moreover, growing evidence suggests that oxidative stress and antioxidant defense mechanisms represent important components of milk quality and may contribute to infant development and health. These advances highlight the importance of investigating not only the nutritional composition but also the antioxidant properties of human milk in women with metabolic disorders [9].

Given the established importance of human milk for infant development and the potential associations between maternal thyroid disease, diabetes, and milk composition, the primary outcome of this study was the assessment of the antioxidant status of human milk, determined by DPPH radical scavenging activity and FRAP. Secondary outcomes included the basic nutritional composition of human milk (fat, protein, carbohydrate, dry matter, and energy content) and cortisol concentration.

## 2. Materials and Methods

### 2.1. Study Group

The study group included 208 breastfeeding women from the Kuyavian-Pomeranian Voivodeship in Poland. Women were recruited via social media and at the Clinic of Obstetrics, Women’s Diseases, and Oncological Gynecology, University Hospital No. 2 named after Dr. Jan Biziel in Bydgoszcz. Participants were recruited through announcements distributed via social media platforms to increase access to potential volunteers. Recruitment was open to all eligible breastfeeding women meeting the predefined inclusion criteria. All interested participants underwent the same screening procedure, and enrollment was based exclusively on the predefined inclusion and exclusion criteria, irrespective of the recruitment channel. The breastfeeding women were divided into three groups: a group of women with type 2 diabetes, a group of women with hypothyroidism, and a control group. The recruitment period for participants ran from January 2019 to December 2023.

The research was approved by the Bioethics Committee of the Nicolaus Copernicus University in Toruń at the Ludwik Rydygier Collegium Medicum in Bydgoszcz (consent no. KB437/2018 and KB121/2019).

All women participating in the study read information about it, completed the questionnaire and provided written informed consent to participate in the study. The questionnaire, developed specifically for this study, consisted of 58 questions. The first part of the questionnaire included questions about age, residence, education, civil status, and professional activity. The subsequent questions collected information on maternal weight, height, gestational age at delivery, parity, medical conditions during pregnancy and lactation, and mode of delivery.

### 2.2. Group Characteristics

Eligible participants for the study included women living in the Kuyavian-Pomeranian Voivodeship (Poland), lactating from the 3rd to the 12th month, supplementing with omega-3 fatty acids (DHA—800 mg, EPA—68 mg), vitamin D (2000 IU), iodine (200 µg), and folic acid (200 µg), and not taking probiotics. Participants had been taking these supplements for at least six months. Women who had used antibiotics in the previous 6 months were excluded. The women did not smoke or drink alcohol. The women breastfed, and the children were not supplemented with formula. Children older than 6 months were given a supplemental diet.

**Figure 1:**
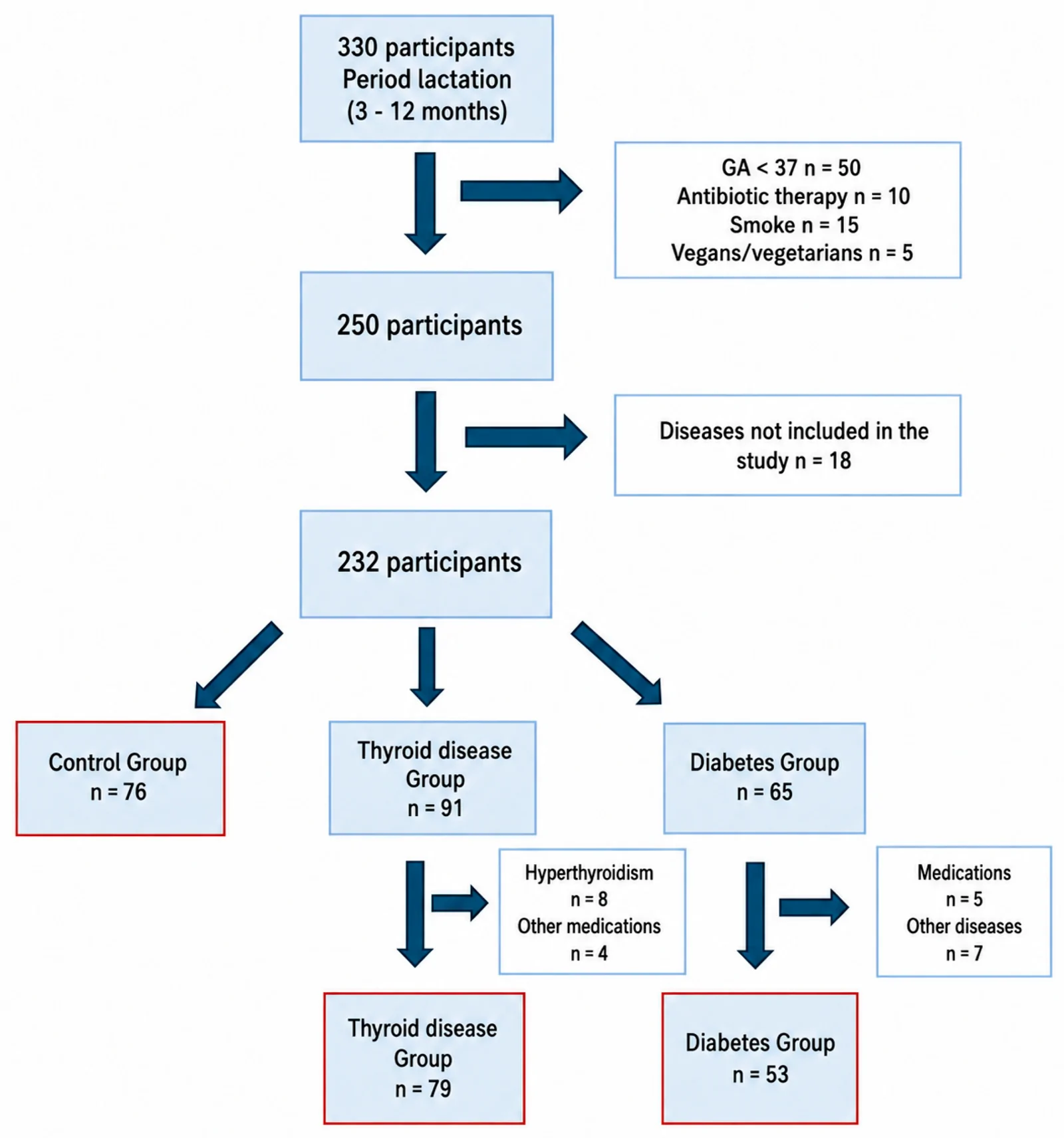
Study participant qualification scheme.

Figure 1 illustrates the participant selection process.

The group of women with thyroid disease included 79 women with hypothyroidism, taking Euthyrox at a dose of 50–100 µg/day, not taking other medications, and without other comorbidities. All patients were metabolically stable, with TSH (0.4–4 mlU/L), FT3 (2.0–4.4 pg/mL), and FT4 (0.7–1.5 ng/dL) levels within the laboratory reference ranges. All patients had been treated for hypothyroidism for at least three years.

The control group included 76 healthy women not taking any medications. None of the women followed any dietary restrictions.

The diabetic group included 53 women with type 2 diabetes. Women with documented type 2 diabetes were enrolled, with an HbA1c (glycated hemoglobin) level in the range of 6–9%, a fasting blood glucose level of ≥126 mg/dL—180 mg/dL, no pharmacotherapy (antidiabetic medication) for at least 6 months prior to the study, and active management of the condition through lifestyle modifications (a low-glycemic index diet and regular physical activity). Women with other comorbidities and those taking medication were excluded.

### 2.3. Materials and Methods Collecting Human Milk

Human milk samples were collected from breastfeeding participants, with a total of 40 mL obtained from each participant over a 24 h period. During the day, the studied group of women expressed milk by hand in four time slots: 06:00–12:00, 12:00–18:00, 18:00–24:00 and 24:00–6:00. The amount of manually expressed milk in each time interval was 10 mL, with 5 mL before the baby latching on and 5 mL after the end of feeding. Each portion of milk was poured into one collective bottle. The sample was stored in a fridge (4 °C) by the woman until it was handed over to the laboratory.

The milk samples were delivered to the laboratory within 24 h of collection, portioned into 2 mL of Eppendorf tubes (MedLab, Warsaw, Poland) and frozen at −20 °C and then −80 °C. The material was stored in such conditions until the parameters described below were determined but not longer than 6 months.

### 2.4. Determination of the Basic Composition of Human Milk

Determination of fat content [g/dL], total protein [g/dL], carbohydrates [g/dL], dry matter [g/dL] and energy value [kcal/dL]. Determination of these parameters was performed in human milk samples using the MIRIS HMATM (MIRIS AB, Uppsala, Sweden) in accordance with the manufacturer’s procedure [HMA]. The limits of detection (LOD) for each component was as follows: fat content—0.11 g/dL, nutritional protein—0.25 g/dL, total protein—0.20 g/dL and carbohydrates—0.35 g/dL.

Before analysis, the human milk samples were heated at 40 °C in a thermostatic bath (BIONOVO, Legnica, Poland) and then homogenized with an MIRIS Sonicator [1.5 s/mL]. Each sample was analyzed in three replicates.

### 2.5. Determination of Cortisol Concentration in Human Milk

The level of cortisol in human milk was determined by enzyme-linked immunosorbent assay (CORTISOL saliva ELISA DiaMetra, Spello, Italy). Cortisol concentration was calculated from a calibration curve (y = 1.66825 + ((0.18995 − 1.66825)/(1 + (x/5.02629)^−0.832414)); R2 = 0.987). The detection limit was 0.12 ng/mL.

### 2.6. Analysis of the Antioxidant Status of Human Milk

Before determination, the samples of human milk were gradually thawed in the refrigerator and then centrifuged for 5 min at 10,000× *g* at 4 °C. The defatted supernatant was transferred into clean test tubes and used for subsequent analysis.

### 2.7. Determination of the Antioxidant Activity of Human Milk Using the DPPH• Radical (2.2-Diphenyl-1-picrylhydrazyl)

The antioxidant activity of human milk was determined using the method described by Atanassov et al. (2011), with minor modifications [10].

### 2.8. Determination in Human Milk Samples

A 100 μM DPPH• solution was prepared by dissolving 4 mg of DPPH• in 100 mL of methanol.

First, 1 mL of prepared DPPH• solution was applied to Eppendorf tubes. 250 μL of human milk was added, vortexed and then incubated for 60 min in the dark at room temperature. Before reading, the samples were centrifuged for 2 min at 1500× *g* (room temperature). Absorbance was measured at a wavelength of 517 nm against a reference test, which was methanol. The control sample was a 100 μM methanol solution of DPPH• whose absorbance was measured at the beginning and end of the experiment.

The results were expressed as the percentage ability of human milk to reduce the DPPH radical, calculated as follows:

$$\%\;\mathrm{inhibition}=(\frac{A-\mathrm{Ab}}{A})\times 100$$

A—control absorbance;

Ab—average absorbance value of the tested human milk.

### 2.9. Validation of the Method

#### 2.9.1. Linearity

The linearity assessment was carried out using a calibration curve based on the absorbance of Trolox at various concentrations. The solutions for obtaining the calibration curve were prepared in the same way, replacing 250 μL of human milk with solutions of Trolox (TEα (+-)-hydroxy-2.5.7.8-tetramethylchromatane-2-carboxylic acid, Sigma Aldrich, Burlington, MA, USA) at concentrations of 10–80 mg/L. The standard curve shows the dependence of the absorbance value of Trolox on its concentration (y = −0.0101x + 0.8161 R2 = 0.99781).

#### 2.9.2. Precision

Precision was determined using the same sample of human milk, with and without the addition of the positive control, in three replicates. Trolox was used at concentrations of 10 mg/L and 40 mg/L. The relative standard deviation was calculated using the following formula:

%RSD = S/X ∗ 100

S—the standard deviation of each measurement;

X—average of the measurements.

#### 2.9.3. LD

The limit of detection was determined on the basis of the standard deviation of the set of signals and the slope of the calibration curve.

LD = 3.3S/b

S—standard deviation, calculated as the s of the results obtained for a series of blank samples;

b—slope of the calibration curve;

Detection limit: 0.1 μg TE/mL.

### 2.10. Determination of the Ability of Human Milk to Reduce Fe (III) Ions

The FRAP method (ability to reduce iron ions) according to Benzie and Strain (1999) was used in the experiment, with modifications [11].

A blank test was prepared, consisting of the FRAP reagent, and 100 μL of human milk samples and standard solutions were used for the determination of the calibration curve. The next step was to apply 50 μL of the sample and 1.5 mL of the FRAP reagent, followed by vortexing and immediate sample absorbance reading (at 0 min) at 593 nm and incubation in a water bath at 37 °C for 4 min. The samples were thoroughly mixed, and the absorbance was measured again at the same wavelength.

$$\mathrm{FRAP}=\frac{\mathrm{sample}\;\mathrm{absorbance}\;\mathrm{change}\;\mathrm{from}\;0\;\min\mathrm{to}\;4\;\min}{\mathrm{standard}\;\mathrm{absorbance}\;\mathrm{change}\;\mathrm{from}\;0\min\mathrm{to}\;4\;\min}\times \mathrm{standard}\;\mathrm{concentration}\times 2\;[\mu M]$$

Ascorbic acid has a constant stoichiometric coefficient of 2.0 in the FRAP test.

*The method was validated in accordance with the validation procedure described in the DPPH method linearity.*

A six-point calibration curve for ascorbic acid (Chempur, Piekary Śląskie, Poland) was established using standard solutions across a concentration range of 100–1000 μM. FRAP concentrations were expressed as the equivalent of ascorbic acid (μM) calculated from the standard curve (y = 0.0006x−0.0392 R2 = 0.9916). The FRAP value is calculated from the following formula:

LD

Detection limit: 2 µM

### 2.11. Statistical Analysis

Statistical analyses were performed using Statistica software, version 13.1 (StatSoft, Kraków, Poland).

The normality of the distribution was verified by the Shapiro–Wilk test. The Shapiro–Wilk test indicated that the analyzed quantitative variables did not follow a normal distribution. The Kruskal–Wallis test was used to analyze multiple independent groups. For significant overall results, pairwise post hoc comparisons of the mean ranks were performed.

The variability of the parameters is presented in the form of a median lower and upper quartile (Q25–Q75).

In order to analyze many factors per variable in the studied groups, multiple linear regression was used. The variability of the parameters is presented in the form of a regression coefficient, the error of the standard regression coefficient, R^2^, adjusted R^2^ and the level of statistical significance *p* < 0.05.

The effect size (r) was calculated using the following formula:

r = Z/√*N*

r = Z/√*N*;

Z—standardized Kruskal–Wallis test statistic;

*N*—sample size.

To assess the correlation between the parameters studied, the Spearman correlation test was used. The Bonferroni correction for correlation was applied by adjusting the significance level based on the total number of variable pairs tested. The number of comparisons was calculated using the following formula:

m = (*N* ∗ (*N* − 1))/2, where *N*—variables

The level of statistical significance was then adjusted as follows:

*p*_new_ = *p*/m, where *p* = 0.05

The results at the level of *p* < 0.001 were considered statistically significant.

The strength of the associations between variables was interpreted according to Guilford’s classification, as follows:

- |rs| < 0.2—no linear relationship.
- 0.2 ≤ |rs| < 0.4—weak dependency.
- 0.4 ≤ |rs| < 0.7—moderate dependence.
- 0.7 ≤ |rs| < 0.9—strong dependency.
- |rs| ≥ 0.9—a very strong dependency.

The correlations that occur are represented by a correlation matrix.

Prior to the multiple linear regression analysis, its underlying assumptions were verified. Linearity, homoscedasticity, and normality of residuals were assessed and confirmed using residual plots. Multicollinearity among the independent variables was evaluated using the Variance Inflation Factor (VIF). All variables met the criteria for acceptable independence, with VIF values remaining well below the standard threshold of 5.0, indicating no significant multicollinearity issues.

## 3. Results

The diabetic group was characterized by an 11.2% higher BMI and a 5% lower gestational age at delivery (GA) compared to the control group (Table 1A). Vaginal delivery was the most common mode of delivery across all study groups. (Table 1B).

### 3.1. Analysis of the Basic Composition and Concentration of Cortisol in Human Milk in the Groups Studied

The basic composition of the milk of women with hypothyroidism differed from that of the control group. Fat levels were 20% higher in the milk of women with hypothyroidism (Table 2). However, in the diabetic group, fat levels were approximately 28% lower compared to the control group. Analysis of cortisol concentration in milk revealed no significant differences between the study groups.

A 13.8% lower reduction in the DPPH radical was observed in breast milk from diabetic women and a 29% lower reduction was observed in breast milk from women with hypothyroidism compared to the control group (Table 2).

### 3.2. Correlation

The results of Spearman’s rank correlation analyses for each study group are presented in Table 3, Table 4 and Table 5. A number of correlations with a low effect size were identified. In the group of women with thyroid disorders, several statistically significant correlations with a moderate effect size were observed. As the concentration of milk dry matter increases, so do the levels of fat (r = 0.719, *p* < 0.001) and total protein (r = 0.412, *p* = 0.001). In addition, the following positive correlations were observed in this group: caloric value–total protein (r = 0.404, *p* = 0.001) and caloric value–fat (r = 0.670, *p* 0.001). In the group of patients with type II diabetes, two statistically significant correlations with a moderate effect were demonstrated. An increase in total protein levels in milk was observed with increasing dry matter content (r = 0.517, *p* = 0.001). In both groups, one statistically significant correlation with a strong effect was observed. As the dry matter content of milk increased, so did its calorific value (r = 0.882; r = 0.943; *p* < 0.001).

To investigate the associations between additional maternal and clinical variables (study group, BMI, GA, age, mode of delivery, duration of lactation, and place of residence) and the analyzed human milk parameters, multiple linear regression analyses were performed (Table 6 and Table 7).

It has been shown that the antioxidant status of human milk (assessed using the DPPH radical) may be associated with not only by the breastfeeding mother’s type 2 diabetes but also, concurrently, with GA. However, no additional influence of other predictors on DPPH levels was observed when taking into account thyroid disorders in breastfeeding women.

Given the exploratory nature of the correlation analyses and the large number of comparisons performed, isolated statistically significant weak correlations should be interpreted cautiously. Such associations may reflect chance findings and should not be considered clinically meaningful without confirmation in independent studies.

## 4. Discussion

Previous studies indicate that hypothyroidism is associated with alterations in circulating hormones, including corticosterone, prolactin, and progesterone, which may contribute to differences in the quality and quantity of human milk production [12]. This may be related to the fact that prolactin promotes the synthesis of milk proteins such as β-casein and α-lactalbumin, and adequate thyroid hormone levels are necessary for milk production in response to prolactin [13]. A subsequent study demonstrated that maternal plasma thyroxine levels are not only positively correlated with milk production but also influence milk protein synthesis [14].

With regard to the composition of human milk, Kivinen et al. (1979) [15] did not observe statistically significant differences in fatty acid composition between women taking TRH and those not taking it. Motil et al. (1994) [16] showed that there was a positive association between thyroxine and triiodothyroxine and the amount of milk produced and the composition of milk at 1, 5, and 12 months postpartum. Tyson et al. (1976) [17] observed that when assessing the weekly weight of infants in groups of mothers treated with TRH and those not treated, there was an increase in infant weight in the first week; however, when assessing the same groups at 12 and 16 weeks, there was no difference in infant weight gain in mothers who received oral tripeptide. The researchers assessed that thyrotropin-releasing hormone administration was not associated with changes in the composition of human milk in terms of percentage protein and fat at birth compared with the control group, who did not receive thyrotropin-releasing hormone. A gradual and significant decline in the mean protein concentration of human milk was also observed between 1 and 12 weeks postpartum [17]. Chen et al. (2018) [18] demonstrated that gestational hypothyroidism in lactating women can alter the whey protein composition of colostrum. The authors also assessed that hypothyroidism increases levels of protein related to the immune system, which may impact maternal and infant health.

In this study, significant differences between the groups in terms of antioxidant capacity were observed in the DPPH assay, whilst FRAP values did not differ significantly between the groups studied. These results should therefore not be interpreted as evidence of a general reduction in the antioxidant status of human milk. Rather, they indicate a lower DPPH radical-scavenging capacity in the milk of women with type 2 diabetes and hypothyroidism compared to the control group. The discrepancy between the DPPH and FRAP results may stem from the different mechanisms of antioxidant activity assessed by these methods and highlights the need to interpret them separately [7,19].

Diseases that may alter the composition of human milk also include diabetes, which has been associated with changes in macronutrient content and selected bioactive components in clinical studies. Available data indicate that gestational diabetes is associated with disturbances in glucose and lipid metabolism [20,21,22], which may affect the composition of human milk. Zhong et al. [23] demonstrated higher triglyceride concentrations in women with GDM, accompanied by changes in the lipid content of colostrum, and a relationship between these changes and higher birth weight of newborns. These findings indicate that metabolic disturbances characteristic of GDM may modify the macronutrient composition of human milk.

The composition of human milk undergoes dynamic changes throughout lactation. In comparison with colostrum, mature human milk contains less protein, whereas the concentrations of fat and lactose tend to increase progressively, as demonstrated in previous studies. These physiological changes should be taken into account when interpreting the results of analyses comparing the composition of milk from women with gestational diabetes and healthy women. Importantly, most available studies on changes in human milk associated with diabetes have been conducted in women with gestational diabetes, rather than in those with a diagnosis of type 2 diabetes. Consequently, these findings provide only an indirect context for the present results and should not be directly applied to women with type 2 diabetes, given the differences in disease duration, underlying metabolic disturbances, and the progression of metabolic processes during the postpartum period. Qin et al. reported that mature milk from women with gestational diabetes mellitus (GDM) may have a higher energy value due to increased lipid and protein content. In contrast, among women with type 2 diabetes in our study, the median energy content of milk was modestly lower than that observed in the control group, although the difference did not reach statistical significance (Table 2). The divergent findings may be related to differences between GDM and type 2 diabetes, as well as to methodological differences and variation in the characteristics of the study populations. This observation may reflect persistent postpartum metabolic changes related to lipid and carbohydrate metabolism. It can be assumed that the process of lactation promotes the gradual normalization of these disorders through increased utilization of energy substrates for milk synthesis, but the mechanisms responsible for this phenomenon have not yet been clearly elucidated [4]. These results are consistent with observations from prospective studies, which have shown that longer breastfeeding duration is associated with a lower risk of developing type 2 diabetes in women with a history of gestational diabetes [23]. Similar findings were also reported by Fanzhu Xue [24], suggesting that lactation may favorably influence the regulation of glucose and lipid metabolism in women with gestational diabetes. Although the biological mechanisms responsible for these effects remain incompletely understood, the increased energy expenditure required for milk synthesis may contribute to the restoration of metabolic homeostasis during the postpartum period.

It should be noted that maternal metabolic disorders are characterized by chronic low-grade inflammation, insulin resistance, and increased oxidative stress, all of which may influence mammary gland metabolism and consequently alter the synthesis and secretion of milk components. Increased production of reactive oxygen species, together with impaired antioxidant defense, may affect mammary epithelial cell function, lipid metabolism, and the transfer of bioactive compounds into human milk. Such processes may, at least in part, account for the differences in milk composition and antioxidant activity identified in our study. Maternal obesity, which frequently coexists with type 2 diabetes, has also been associated with changes in human milk composition through inflammatory pathways involving adipokines, cytokines, and altered insulin signaling. These metabolic disturbances may influence both macronutrient composition and the concentration of bioactive molecules in breast milk. Therefore, maternal metabolic status may be associated with differences in human milk composition. [4].

In the unadjusted between-group comparisons, milk fat content was significantly higher in women with hypothyroidism and lower in women with type 2 diabetes compared with the control group. However, these associations were no longer statistically significant after adjustment for additional maternal and clinical variables in the multiple regression analyses (Table 6 and Table 7). Specifically, neither type 2 diabetes (*p* = 0.933) nor hypothyroidism (*p* = 0.599) was independently associated with milk fat content in the adjusted models.

These findings suggest that the differences observed in the unadjusted analyses may be partly explained by other maternal or clinical characteristics rather than by the underlying diseases alone. Therefore, the observed differences in milk fat content should be interpreted cautiously and should not be considered evidence of an independent effect of maternal diabetes or hypothyroidism on human milk composition.

Available evidence suggests that longer breastfeeding duration may be associated with beneficial metabolic effects in women with a history of gestational diabetes, including a gradual improvement in metabolic parameters and a potentially lower risk of later disturbances in carbohydrate metabolism. However, it should be emphasized that the current evidence is mainly observational; therefore, confirmation of a causal relationship requires further prospective studies of high methodological quality.

### Limitations and Strengths of the Work

To our knowledge, this is the first study to characterize both the composition and antioxidant status of human milk in women with type 2 diabetes and hypothyroidism and to compare these parameters with those observed in healthy women. This study has several limitations that should be considered when interpreting the findings. First, the cross-sectional observational design does not allow causal relationships to be established between maternal metabolic disorders and changes in human milk composition. Furthermore, the significant differences in milk fat content observed in unadjusted comparisons were not confirmed in the adjusted multiple regression models. This finding highlights the potential influence of maternal and clinical covariates and underscores the importance of interpreting the unadjusted group differences cautiously. Second, participants were recruited from a single geographic region, which may limit the generalizability of the results to other populations. Third, although participants were classified according to their medical diagnoses, comprehensive biochemical assessment of disease control throughout the lactation period was not available for all participants, which may have influenced milk composition. Fourth, maternal dietary intake, which is known to affect certain components of human milk, was not assessed. In addition, infant growth, body composition, and developmental outcomes were not assessed, making it difficult to determine the clinical relevance of the observed differences in milk composition. An additional limitation concerns the use of a 24 h pooled milk sample for cortisol measurement; future studies should consider collecting a separate morning sample to account for the diurnal variation in cortisol levels.

Finally, as data on the composition of breast milk in women with a diagnosis of type 2 diabetes are limited, part of the discussion is based on studies conducted among women with gestational diabetes; therefore, these comparisons should be considered indirect and interpreted with caution.

## 5. Conclusions

This observational cross-sectional study identified differences in selected characteristics of human milk among women with type 2 diabetes, hypothyroidism, and healthy controls. In the unadjusted analyses, significant between-group differences were observed in milk fat content and DPPH radical-scavenging activity. However, after adjustment for maternal and clinical covariates, neither type 2 diabetes nor hypothyroidism was independently associated with milk fat content. Significant between-group differences in DPPH radical-scavenging activity were observed, whereas FRAP values did not differ significantly between the groups. These findings suggest assay-specific differences in antioxidant activity rather than a generalized alteration in the antioxidant status of human milk. The findings should be interpreted cautiously, as unadjusted between-group differences do not necessarily indicate independent disease-related associations. Moreover, the clinical significance of these observations remains uncertain because infant growth, body composition, and developmental outcomes were not assessed.

Furthermore, the observed associations may have been influenced by maternal BMI, stage of lactation, dietary factors, disease control, and other unmeasured maternal or environmental variables. Given the observational nature of the study, no conclusions regarding causality can be drawn. These findings should therefore be considered exploratory and require confirmation in larger prospective studies incorporating comprehensive assessments of maternal metabolic status, human milk composition, and infant health outcomes. Importantly, our findings do not support restricting or discouraging breastfeeding in women with hypothyroidism or type 2 diabetes, as breastfeeding remains the recommended form of infant feeding in these groups.

## Figures and Tables

**Table 1:** (**A**) Characteristics of the study groups. (**B**) Breakdown of the study groups according to additional predictors.

(A)										
	Diabetes	Control		Thyroid Disease	Control		Diabetes/Thyroid Disease	Effect Size		
	N = 53	N = 76	*p*	N = 79	N = 76	*p*	*p*	r Diabetes/Control	r Thyroid Disease/Control	r Diabetes/Thyroid Disease
Age [years] (Me; Q25–Q75	32 29–34	30 28–32	0.916	30 28–33	30 28–32	0.912	0.455	0.126	0.037	0.082
BMI [kg/m^2^] (Me; Q25–Q75)	25.64 20.97–27.20	22.76 21.1–24.86	0.015	22.51 20.17–25.29	22.76 21.1–24.86	0.816	0.187	0.164	0.088	0.244
GA (Me; Q25–Q75)	38 37–40	40 39–41	0.008	40 39–40	40 39–41	0.309	<0.001	0.379	0.119	0.261
(B)										
Variable		Control Group (G1)		Diabetes Group (G2)		Thyroid Diseases (G3)		*p* [G1/G2]	*p* [G1/G3]	*p* [G2/G3]
Delivery type		N (%)								
Vaginal delivery		40 (53%)		35 (66%)		55 (70%)		0.576	0.990	0.665
Cesarean section		36 (47%)		18 (34%)		24 (30%)		>0.999	0.617	0.634
Place of residence		N (%)								
City center		44 (58%)		28 (53%)		40 (51%)		0.723	0.508	0.862
The outskirts of the city		32 (42%)		25 (47%)		39 (49%)		0.720	0.999	0.937
Maternal Parity		N (%)								
Primiparous		43 (56%)		26 (49%)		42 (53%)		0.551	0.999	0.812
Multiparous		33 (44%)		27 (51%)		37 (47%)		0.560	0.848	0.723

(**A**) N—size; BMI—body mass index [kg/m^2^]; GA—gestational age at delivery; Me—median; Q25–Q75—Lower and Upper Quartile; *p*—level of statistical significance (Kruskal–Wallis test).

**Table 2:** Analysis of cortisol concentration, basic composition, and antioxidant status of human milk in the study groups.

	Diabetes	Control	*p*	Thyroid Diseases	Control	*p*	Diabetes/Thyroid Diseases	Effect Size		
	N = 53	N = 76		N = 79	N = 76		*p*	r Diabetes/Control	r Thyroid Disease/Control	r Diabetes/Thyroid Disease
Fats [g/dL] (Me; Q25–Q75)	2.2 1.6–3.4	3.05 1.6–4.55	0.001	3.8 3.8–5.0	3.05 1.6–4.55	0.003	0.089	0.191	0.261	0.433
Total protein [g/dL] (Me; Q25–Q75)	1.2 1.1–1.4	1.2 1.1–1.3	0.629	1.2 1.1–1.3	1.2 1.1–1.3	0.999	0.816	0.098	0.015	0.109
Carbohydrates [g/dL] (Me; Q25–Q75)	7.9 7.5–8.3	7.9 7.55–8.1	0.235	7.8 7.4–8.2	7.9 7.55–8.1	0.785	0.999	0.070	0.091	0.153
Dry matter [g/dL] (Me; Q25–Q75)	12.7 12.2–14	13.2 12.1–14.65	0.991	13.2 12–14.5	13.2 12.1–14.65	0.999	>0.999	0.053	0.001	0.059
Caloric value [kcal/dL] (Me; Q25–Q75)	70 64–79	72.5 62.5–86.5	0.999	72 64–85	72.5 62.5–86.5	0.990	>0.999	0.059	0.001	0.053
Cortisol [ng/mL] (Me; Q25–Q75)	9.77 5.34–15.95	8.44 4.52–11.8	0.649	8.7 5.74–11.61	8.44 4.52–11.8	0.991	0.649	0.109	0.028	0.133
FRAP [µM] (Me; Q25–Q75)	814.31 408.47–1253.74	819.35 502.24–1147.93	0.992	781.23 555.64–1035.58	819.35 502.24–1147.93	0.928	>0.999	0.022	0.013	0.035
DPPH [%] (Me; Q25–Q75)	50.74 45.99–73.39	63.08 55.6–72.93	0.018	52.63 47.05–63.94	63.08 55.6–72.93	0.001	>0.999	0.059	0.235	0.223

N—size; Me—median; Q25–Q75—lower and upper quartile; *p*—level of statistical significance (Kruskal–Wallis test); FRAP—iron ion reduction ability; DPPH—radical 2.2-diphenyl-1-picrylhydrasylum.

**Table 3:** Correlations of the Spearman rank order for the control group.

Variable	GA	Age	BMI	Cortisol	DPPH	FRAP	Fat	Total Protein	Carbohydrates	Dry Matter	Caloric Value
GA	1.000	0.013	0.042	0.111	−0.183	−0.189	−0.191	−0.029	0.100	−0.168	−0.184
Age	0.013	1.000	0.057	0.042	0.042	−0.063	0.083	−0.047	−0.001	0.029	−0.009
BMI	0.042	0.057	1.000	−0.025	−0.008	0.112	−0.074	0.018	0.017	0.013	−0.007
Cortisol	0.111	0.042	−0.025	1.000	−0.283	−0.114	−0.175	0.046	0.092	0.018	0.041
DPPH	−0.183	0.042	−0.008	−0.283	1.000	0.413	0.116	0.071	−0.076	0.053	0.132
FRAP	−0.189	−0.063	0.112	−0.114	0.413	1.000	0.034	0.135	−0.113	−0.089	−0.078
Fat	−0.191	0.083	−0.074	−0.175	0.116	0.034	1.000	0.133	0.035	0.718	0.693
Total protein	−0.029	−0.047	0.018	0.046	0.071	0.135	0.133	1.000	0.089	0.319	0.258
Carbohydrates	0.100	−0.001	0.017	0.092	−0.076	−0.113	0.035	0.089	1.000	0.395	0.201
Dry matter	−0.168	0.029	0.013	0.018	0.053	−0.089	0.718	0.319	0.395	1.000	0.891

This table show correlation coefficients; those marked in red indicate *p* < 0.001. BMI—body mass index; FRAP—iron ion reduction capacity [μM]; DPPH—2.2-diphenyl-1-picrylhydrazyl radical [% inhibition]; cortisol—concentration in ng/mL; Fat, total protein, carbohydrates, and dry matter—content in human milk in g/100 mL; energy value—content in kcal/100 mL.

**Table 4:** Correlations of the Spearman rank order for the thyroid diseases group.

Variable	GA	Age	BMI	Cortisol	DPPH	FRAP	Fat	Total Protein	Carbohydrates	Dry Matter	Caloric Value
GA	1.000	0.015	−0.176	−0.071	−0.433	−0.163	0.050	−0.006	0.048	−0.094	−0.114
Age	0.015	1.000	0.214	−0.045	0.047	−0.064	−0.015	0.014	−0.146	−0.024	−0.047
BMI	−0.176	0.214	1.000	−0.242	0.096	0.237	−0.249	0.052	−0.030	−0.137	−0.116
Cortisol	−0.071	−0.045	−0.242	1.000	−0.182	−0.046	−0.004	0.218	0.134	0.171	0.250
DPPH	−0.433	0.047	0.096	−0.182	1.000	0.552	0.128	0.138	−0.171	0.130	0.207
FRAP	−0.163	−0.064	0.237	−0.046	0.552	1.000	−0.004	0.095	−0.228	−0.089	0.021
Fat	0.050	−0.015	−0.249	−0.004	0.128	−0.004	1.000	0.143	−0.007	0.719	0.670
Total protein	−0.006	0.014	0.052	0.218	0.138	0.095	0.143	1.000	0.039	0.412	0.404
Carbohydrates	0.048	−0.146	−0.030	0.134	−0.171	−0.228	−0.007	0.039	1.000	0.411	0.248
Dry matter	−0.094	−0.024	−0.137	0.171	0.130	−0.089	0.719	0.412	0.411	1.000	0.882

This table show correlation coefficients; those marked in red indicate *p* < 0.001, BMI—body mass index; FRAP—iron ion reduction capacity [μM]; DPPH—2.2-diphenyl-1-picrylhydrazyl radical [% inhibition]; cortisol—concentration in ng/mL; Fat, total protein, carbohydrates, and dry matter—content in human milk in g/100 mL; energy value—content in kcal/100 mL.

**Table 5:** Correlations of the Spearman rank order for the diabetes group.

Variable	GA	Age	BMI	Cortisol	DPPH	FRAP	Fat	Total Protein	Carbohydrates	Dry Matter	Caloric Value
GA	1.000	−0.017	−0.208	−0.210	−0.248	0.090	0.255	−0.236	−0.149	−0.117	−0.074
Age	−0.017	1.000	0.003	0.063	0.086	−0.027	−0.138	0.026	0.111	0.005	−0.041
BMI	−0.208	0.003	1.000	0.046	−0.018	−0.184	−0.073	0.027	−0.021	0.173	0.203
Cortisol	−0.210	0.063	0.046	1.000	0.084	−0.193	−0.269	0.402	0.067	0.317	0.292
DPPH	−0.248	0.086	−0.018	0.084	1.000	0.015	0.083	0.082	−0.075	0.186	0.209
FRAP	0.090	−0.027	−0.184	−0.193	0.015	1.000	0.187	−0.186	−0.138	−0.189	−0.083
Fat	0.255	−0.138	−0.073	−0.269	0.083	0.187	1.000	0.106	−0.180	0.235	0.326
Total protein	−0.236	0.026	0.027	0.402	0.082	−0.186	0.106	1.000	−0.072	0.517	0.484
Carbohydrates	−0.149	0.111	−0.021	0.067	−0.075	−0.138	−0.180	−0.072	1.000	0.263	0.027
Dry matter	−0.117	0.005	0.173	0.317	0.186	−0.189	0.235	0.517	0.263	1.000	0.943

This table show correlation coefficients; those marked in red indicate *p* < 0.001, BMI—body mass index; FRAP—iron ion reduction capacity [μM]; DPPH—2.2-diphenyl-1-picrylhydrazyl radical [% inhibition]; cortisol—concentration in ng/mL; Fat, total protein, carbohydrates, and dry matter—content in human milk in g/100 mL; energy value—content in kcal/100 mL.

**Table 6:** Multiple linear regression analysis of associations between selected variables and human milk parameters (including type 2 diabetes and a control group).

Variable	Predictor	B ± SE	95%Cl	*p*
Fat	R = 0.099 R^2^ = 0.009 R^2^_adj_ = −0.020			
	Diabetes vs control	0.006 ± 0.078	−0.147; 0.159	0.933
	Delivery Type	−0.001 ± 0.073	−0.144; 0.142	0.987
	Age	−0.126 ± 0.075	−0.273; 0.021	0.093
	BMI	0.069 ± 0.074	−0.076; 0.214	0.382
	GA	−0.011 ± 0.074	−0.156; 0.134	0.888
	Place of Residence	0.076 ± 0.074	−0.069; 0.221	0.302
	Maternal Parity	0.089 ± 0.069	−0.046; 0.224	0.197
	Lactation Time	0.254 ± 0.069	0.119–0.389	0.001
Total protein	R = 0.221 R^2^ = 0.049 R^2^_adj_ = 0.018			
	Diabetes vs control	0.078 ± 0.076	−0.071; 0.227	0.305
	Delivery Type	0.125 ± 0.076	−0.024; 0.274	0.100
	Age	−0.052 ± 0.072	−0.193; 0.089	0.472
	BMI	0.087 ± 0.073	−0.056; 0.230	0.227
	GA	−0.074 ± 0.076	−0.223; 0.075	0.333
	Place of Residence	0.022 ± 0.072	−0.119; 0.163	0.757
	Maternal Parity	0.125 ± 0.069	−0.010; 0.260	0.070
	Lactation Time	0.098 ± 0.077	−0.053; 0.249	0.154
Carbohydrates	R = 0.107 R^2^ = 0.011 R^2^_adj_ = 0.002			
	Diabetes vs control	0.021 ± 0.078	−0.132; 0.174	0.787
	Delivery Type	−0.075 ± 0.073	−0.218; 0.068	0.305
	Age	−0.049 ± 0.074	−0.194; 0.096	0.497
	BMI	0.057 ± 0.074	−0.088; 0.202	0.439
	GA	0.007 ± 0.077	−0.144; 0.158	0.927
	Place of Residence	0.022 ± 0.074	−0.123; 0.167	0.783
	Maternal Parity	0.047 ± 0.069	−0.088; 0.182	0.256
	Lactation Time	0.020 ± 0.071	−0.119; 0.159	0.562
Dry matter	R = 0.128 R^2^ = 0.016 R^2^_adj_ = 0.007			
	Diabetes vs control	−0.009 ± 0.079	−0.164; 0.146	0.905
	Delivery Type	−0.055 ± 0.073	−0.198; 0.088	0.452
	Age	0.049 ± 0.073	−0.094; 0.192	0.501
	BMI	0.061 ± 0.074	−0.084; 0.206	0.384
	GA	−0.066 ± 0.077	−0.217; 0.085	0.391
	Place of Residence	0.010 ± 0.074	−0.135; 0.155	0.887
	Maternal Parity	0.085 ± 0.069	−0.050; 0.220	0.258
	Lactation Time	0.125 ± 0.071	−0.014; 0.264	0.141
Caloric value	R = 0.116 R^2^ = 0.013 R^2^_adj_ = 0.002			
	Diabetes vs control	−0.028 ± 0.078	−0.181; 0.125	0.720
	Delivery Type	−0.028 ± 0.073	−0.171; 0.115	0.701
	Age	0.012 ± 0.073	−0.131; 0.155	0.871
	BMI	0.079 ± 0.074	−0.066; 0.224	0.281
	GA	−0.071 ± 0.079	−0.226; 0.084	0.365
	Place of Residence	0.020 ± 0.073	−0.123; 0.163	0.701
	Maternal Parity	0.054 ± 0.071	−0.085; 0.193	0.524
	Lactation Time	0.101 ± 0.069	−0.034; 0.236	0.129
Cortisol	R = 0.163 R^2^ = 0.027 R^2^_adj_ = 0.018			
	Diabetes vs control	0.152 ± 0.078	−0.001; 0.305	0.051
	Delivery Type Age	−0.025 ± 0.073	−0.168; 0.118	0.726
	BMI	−0.034 ± 0.073	−0.177; 0.109	0.647
	GA	−0.004 ± 0.077	−0.155; 0.147	0.958
	Place of Residence	−0.045 ± 0.073	−0.188; 0.098	0.535
	Maternal Parity	0.032 ± 0.075	−0.115; 0.179	0.528
	Lactation Time	0.065 ± 0.071	−0.074; 0.204	0.257
FRAP	R = 0.200 R^2^ = 0.040 R^2^_adj_ = 0.009			
	Diabetes vs control	−0.105 ± 0.077	−0.256; 0.046	0.173
	Delivery Type	0.072 ± 0.072	−0.069; 0.213	0.320
	Age	−0.002 ± 0.072	−0.143; 0.139	0.786
	BMI	0.064 ± 0.073	−0.079; 0.207	0.379
	GA	−0.020 ± 0.076	−0.169; 0.129	0.786
	Place of Residence	0.072 ± 0.072	−0.069; 0.213	0.312
	Maternal Parity	0.035 ± 0.071	−0.104; 0.174	0.621
	Lactation Time	0.102 ± 0.079	−0.053; 0.257	0.252
DPPH	R = 0.236 R^2^ = 0.055 R^2^_adj_ = 0.025			
	Diabetes vs control	0.147 ± 0.072	0.006; 0.288	0.042
	Delivery Type	0.085 ± 0.071	−0.054; 0.224	0.236
	Age	0.028 ± 0.072	−0.113; 0.169	0.687
	BMI	0.031 ± 0.072	−0.110; 0.172	0.663
	GA	−0.164 ± 0.076	−0.313; −0.015	0.031
	Place of Residence	−0.011 ± 0.076	−0.160; 0.138	0.882
	Maternal Parity	0.067 ± 0.073	−0.076; 0.210	0.158
	Lactation Time	0.121 ± 0.076	−0.028; 0.270	0.098

B—regression coefficient; SE—standard error from the regression coefficient; 95% Cl—95% confidence level; red—*p*—statistical significance level < 0.050 (red).

**Table 7:** Multiple linear regression analysis of associations between selected variables and human milk parameters (including thyroid diseases and a control group).

Variable	Predictor	B ± SE	95%Cl	*p*
Fat	R = 0.106 R^2^ = 0.011 R^2^_adj_ = −0.029			
	Thyroid disease vs control	−0.038 ± 0.071	−0.177; 0.101	0.599
	Delivery Type	−0.001 ± 0.073	−0.144; 0.142	0.982
	Age	0.066 ± 0.074	−0.079; 0.211	0.352
	BMI	−0.013 ± 0.072	−0.154; 0.128	0.864
	GA	−0.013 ± 0.074	−0.158; 0.132	0.324
	Place of Residence	0.073 ± 0.074	−0.072; 0.218	0.866
	Maternal Parity	0.025 ± 0.071	−0.114; 0.164	0.754
	Lactation Time	0.085 ± 0.076	−0.064; 0.234	0.251
Total protein	R = 0.214 R^2^ = 0.044 R^2^_adj_ = 0.014			
	Thyroid disease vs control	−0.028 ± 0.071	−0.167; 0.111	0.687
	Delivery Type	0.162 ± 0.071	0.023; 0.301	0.024
	Age	−0.047 ± 0.073	−0.190; 0.096	0.511
	BMI	0.095 ± 0.073	−0.048; 0.238	0.101
	GA	−0.099 ± 0.072	−0.240; 0.042	0.169
	Place of Residence	0.014 ± 0.072	−0.127; 0.155	0.849
	Maternal Parity	0.125 ± 0.074	−0.020; 0.270	0.125
	Lactation Time	0.256 ± 0.079	0.101; 0.411	0.068
Carbohydrates	R = 0.124 R^2^ = 0.015 R^2^_adj_ = −0.025			
	Thyroid disease vs control	0.065 ± 0.072	−0.076; 0.206	0.371
	Delivery Type	−0.072 ± 0.073	−0.215; 0.071	0.326
	Age	−0.047 ± 0.073	−0.190; 0.096	0.513
	BMI	0.061 ± 0.074	−0.084; 0.206	0.402
	GA	−0.001 ± 0.071	−0.140; 0.138	0.981
	Place of Residence	0.025 ± 0.073	−0.118; 0.168	0.326
	Maternal Parity	0.022 ± 0.073	−0.121; 0.165	0.425
	Lactation Time	0.041 ± 0.074	−0.104; 0.186	0.258
Dry matter	R = 0.130 R^2^ = 0.017 R^2^_adj_ = −0.020			
	Thyroid disease vs control	0.021 ± 0.072	−0.120; 0.162	0.766
	Delivery Type	−0.055 ± 0.073	−0.198; 0.088	0.449
	Age	0.048 ± 0.071	−0.091; 0.187	0.503
	BMI	0.063 ± 0.073	−0.080; 0.206	0.384
	GA	−0.064 ± 0.072	−0.205; 0.077	0.381
	Place of Residence	0.012 ± 0.073	−0.131; 0.155	0.862
	Maternal Parity	0.035 ± 0.072	−0.106; 0.176	0.457
	Lactation Time	0.088 ± 0.074	−0.057; 0.233	0.102
Caloric value	R = 0.113 R^2^ = 0.012 R^2^_adj_ = −0.028			
	Thyroid disease vs control	−0.004 ± 0.072	−0.145; 0.137	0.948
	Delivery Type	−0.030 ± 0.072	−0.171; 0.111	0.680
	Age	0.009 ± 0.071	−0.130; 0.148	0.892
	BMI	0.075 ± 0.073	−0.068; 0.218	0.303
	GA	−0.061 ± 0.073	−0.204; 0.082	0.405
	Place of Residence	0.028 ± 0.074	−0.117; 0.173	0.694
	Maternal Parity	0.045 ± 0.073	−0.098; 0.188	0.524
	Lactation Time	0.087 ± 0.074	−0.058; 0.232	0.127
Cortisol	R = 0.141 R^2^ = 0.019 R^2^_adj_ = −0.021			
	Thyroid disease vs control	−0.015 ± 0.072	−0.156; 0.126	0.112
	Delivery Type	−0.017 ± 0.073	−0.160; 0.126	0.809
	Age	0.008 ± 0.073	−0.135; 0.151	0.908
	BMI	−0.021 ± 0.070	−0.158; 0.116	0.775
	GA	−0.053 ± 0.072	−0.194; 0.088	0.455
	Place of Residence	−0.066 ± 0.074	−0.211; 0.079	0.366
	Maternal Parity	0.045 ± 0.072	−0.096; 0.186	0.405
	Lactation Time	0.087 ± 0.074	−0.058; 0.232	0.208
FRAP	R = 0.236 R^2^ = 0.055 R^2^_adj_ = 0.025			
	Thyroid disease vs control	−0.002 ± 0.072	−0.143; 0.139	0.968
	Delivery Type	0.064 ± 0.071	−0.075; 0.203	0.240
	Age	0.028 ± 0.071	−0.111; 0.167	0.695
	BMI	0.030 ± 0.070	−0.107; 0.167	0.674
	GA	−0.160 ± 0.072	−0.301; −0.019	0.025
	Place of Residence	0.148 ± 0.072	0.007; 0.289	0.041
	Maternal Parity	0.088 ± 0.071	0.051; 0.227	0.289
	Lactation Time	0.125 ± 0.072	−0.016; 0.266	0.084
DPPH	R = 0.315 R^2^ = 0.099 R^2^_adj_ = 0.071			
	Thyroid disease vs control	0.263 ± 0.069	0.128; 0.398	0.001
	Delivery Type	0.071 ± 0.069	−0.064; 0.206	0.314
	Age	−0.007 ± 0.069	−0.142; 0.128	0.921
	BMI	0.061 ± 0.071	−0.078; 0.200	0.385
	GA	0.008 ± 0.068	−0.125; 0.141	0.898
	Place of Residence	0.179 ± 0.070	0.042; 0.316	0.314
	Maternal Parity	0.054 ± 0.071	−0.085; 0.193	0.405
	Lactation Time	0.158 ± 0.069	0.023; 0.293	0.075

B—regression coefficient; SE—standard error from the regression coefficient; 95% Cl—95% confidence level; red—*p*—statistical significance level < 0.050.

## Data Availability

The data presented in this study are available upon request from the corresponding author due to participant privacy and confidentiality and ethical approvals.

## Abbreviations

BMI	Body Mass Index
DPPH	2,2-diphenyl-1-picrylhydrazyl radical reduction method
FRAP	iron ion reduction ability
GAE	gallic acid
GA	gestational age
*p*	level of statistical significance
TE	Trolox
